# Impact of Infant Feeding Mode on Gut Microbiome Composition in Early Life: Evidence from an Egyptian Cohort

**DOI:** 10.1038/s41598-026-66403-6

**Published:** 2026-08-21

**Authors:** Shorok ELHindi, Salah Abdalla, Marwa M. Azab, Mahmoud M. Bendary

**Affiliations:** 1 Department of microbiology and immunology, Faculty of pharmacy, Port-Said University, Port-Said, Egypt; 2 Department of microbiology and immunology, Faculty of pharmacy, Suez Canal University, Ismailia, Egypt

**Keywords:** Gastroenterology, Microbiology

## Abstract

The infant gut microbiome develops rapidly in early life, shaping immune maturation and metabolic pathways. Data from Middle Eastern populations remain limited. We studied Egyptian infants aged 1 to 6 months to assess feeding related differences in gut microbiome composition. Stool samples were collected and the gut microbiota was profiled using 16S rRNA gene sequencing targeting the V3–V4 region. Sequence processing, denoising, and taxonomic assignment were performed in QIIME2. Differentially abundant taxa between feeding groups were identified using LEfSe. Alpha and beta diversity, and co-occurrence networks were analyzed. Feeding mode was the dominant factor shaping microbiome composition and diversity. Artificial feeding showed *Actinobacteria* dominance, near absence of *Bacteroidetes*, and lowest diversity. Breastfeeding was associated with a more balanced phylum distribution, detectable *Bacteroidetes*, increased *Proteobacteria*, and significantly higher richness and diversity. Mixed feeding showed the highest alpha diversity and a *Firmicutes* dominated profile, consistent with a transitional microbial state. Beta diversity clearly separated breastfed from artificially fed infants, with mixed feeding intermediate. Genus level signatures differed by feeding mode. Artificial feeding enriched *Bifidobacterium*. Breastfeeding enriched facultative anaerobes including *Escherichia Shigella* and *Enterobacter*. Mixed feeding enriched fermentative genera such as *Subdoligranulum* and *Eubacterium*. Network analysis revealed structured co-variation among anaerobic commensals in breastfed infants and denser opportunistic connectivity in artificially fed infants. Feeding mode strongly influences gut microbiome development in Egyptian infants. Exclusive breastfeeding is associated with greater microbial richness and broader ecological structure, whereas formula feeding is linked to reduced diversity and a narrower community profile. Mixed feeding reflects an intermediate configuration. These findings support breastfeeding promotion for optimal early immune development.

## Introduction

Gastrointestinal infections in infants such as rotavirus gastroenteritis, norovirus infection, enteropathogenic *Escherichia coli* diarrhea, *Salmonella* gastroenteritis, *Campylobacter* enteritis, and *Giardia* intestinalis infection remain common causes of illness and can lead to diarrhea, dehydration, poor growth, and hospital admission^1,2^. The gut microbiota is important because it supports the gut barrier, limits pathogen growth by using available nutrients and binding sites and guides immune responses that help infants prevent and clear enteric infections. The human gut microbiome forms at birth and develops fast during infancy, with trillions of bacteria, archaea, fungi, and viruses building a community that changes with key early life exposures such as delivery conditions, feeding, antibiotics, and the home environment. This early community helps the infant use nutrients, shapes immune training and tolerance, and lowers pathogen success, so it can influence infection risk and inflammatory responses during a period when the immune system is still learning what is safe and what is harmful^3^. Microbes also support gut stability through specific functions that the infant cannot do alone, including short chain fatty acid production, vitamin synthesis, immune signal tuning, and reinforcement of the epithelial barrier that limits microbial translocation and helps prevent chronic inflammation^4^. Because the microbiome and host systems mature together, the early postnatal period acts as a sensitive window where shifts in colonization can redirect long term trajectories in metabolism, immunity, and neurodevelopment, which makes early life microbiome research important for prevention and for designing feeding and clinical strategies that target root causes rather than late outcomes^5,6^.

The newborn gut contains very few microbes at birth, and colonization starts right away through contact with the mother and the surrounding environment during delivery and early feeding^7,8^. Infants born vaginally often acquire microbes linked to the maternal vaginal and fecal microbiota, with taxa commonly reported such as *Lactobacillus*,* Prevotella*, and *Bifidobacterium*, while cesarean delivered infants more often show early enrichment of skin and hospital associated genera such as *Staphylococcus* and *Enterococcus*^9^. Evidence also shows that delivery mode, antibiotic exposure, and gestational age can shift the timing and direction of early microbial succession^10^. In the first stage, facultative anaerobes such as *Escherichia* and *Enterococcus* reduce oxygen levels in the gut, which supports the later expansion of strict anaerobes including *Bifidobacterium* and *Bacteroides* that tend to dominate later in infancy^11,12^. When this sequence is disrupted, for example by cesarean delivery or antibiotics, microbiome maturation may slow and this may raise the risk of immune imbalance later in life^13,14^.

Many factors shape the infant gut microbiota, and they act from pregnancy through weaning. Delivery mode sets early exposure since vaginal birth tends to transfer maternal vaginal and fecal microbes while cesarean birth increases skin and hospital associated microbes and intrapartum antibiotics can further shift these patterns^8,15^. Gestational age also matters because preterm infants often show delayed establishment of strict anaerobes such as *Bifidobacterium* and *Bacteroides* and higher levels of opportunistic taxa including *Enterobacteriaceae* and *Enterococcus*^11^. Antibiotic exposure in the mother or infant can reduce diversity and disrupt normal succession with effects that may persist beyond the treatment window^6^. Maternal factors also contribute through the maternal gut and maternal diet BMI metabolic health infections and probiotic use which can change what the infant encounters and what thrives^16^.The living context then adds variation through urban or rural setting hygiene water quality household crowding siblings pets daycare season and geography which all change microbial exposure and infection pressure^16,17^. Moreover, the timing and type of solid food introduction and weaning shift the microbiota toward a more adult like pattern while common infant medications can also alter community structure^16^. The most important factor affecting infant gut microbiome is feeding type, which acts as a major driver because human milk provides substrates and immune factors that select specific early life communities while formula feeding can shift composition and has been linked with higher levels of some opportunistic taxa and higher antibiotic resistance gene load in some cohorts^6,18^.

Feeding mode shows the strongest and most consistent effect on infant gut microbiota in early life^19,20^. Human breast milk provides nutrients plus bioactive factors such as immunoglobulins cytokines antimicrobial peptides and living bacteria that shape gut colonization^21^. A key driver is human milk oligosaccharides which the infant cannot digest but specific microbes can use, and this supports the growth of *Bifidobacterium* and *Lactobacillus*^12,22^. These taxa convert these substrates into short chain fatty acids including acetate and butyrate which support barrier function tune immune tolerance and limit pathogen growth^23^. Breastfeeding and formula feeding provide different microbial exposures and substrates, so they can shape gut colonization through differences in bioactive factors and oligosaccharides^9^. Breast milk components tend to support taxa adapted to human milk use, while formula creates a different ecological niche, and mixed feeding can combine signals from both and act as a transitional state. These feeding driven shifts can also influence microbial functions such as short chain fatty acid production and other metabolic pathways relevant to gut barrier and immune signaling^4^. Formula innovations that add prebiotics probiotics or synthetic human milk oligosaccharides aim to partially mimic some breast milk driven effects on the microbiome^22^.

Geography and environment shape infant microbiome assembly alongside feeding, through differences in diet, hygiene, antibiotic exposure, and local product formulations^20,24^. Population studies report broad regional patterns, with many Western cohorts showing higher *Bacteroides* and *Bifidobacterium* and many African and Asian cohorts showing higher *Firmicutes* and *Proteobacteria*, likely linked to environmental exposure, climate, and dietary customs^5,15^.

In Egypt, context factors such as climate, hygiene variation, high cesarean delivery rates, and region-specific formula composition may further shape early colonization, yet published data on Egyptian neonates remain limited^6,8^. Therefore, it is important to compare the infant gut microbiome across natural lactation, artificial lactation, and mixed lactation in an Egyptian cohort.

This study adds novelty because it treats natural lactation, artificial lactation, and mixed lactation as three distinct exposures in Egyptian infants, while much of the existing infant feeding microbiome literature compares only exclusive breastfeeding versus exclusive formula and comes mainly from cohorts in Europe, North America, and parts of Asia, and many studies either exclude mixed fed infants or merge them into broader categories even though mixed feeding is common in real settings^23^.

It also addresses a clear regional gap because African populations remain underrepresented in microbiome studies and reference resources, which limits how well findings from other regions generalize to Egypt where climate, household exposures, health care practices, antibiotic use patterns, and local formula products can differ^25^, so generating Egypt specific data reduces reliance on assumptions and supports locally valid interpretation of how feeding mode relates to early gut microbial development^26,27^.Given the established role of breastfeeding in reinforcing gut barrier integrity and the documented association of cesarean delivery and formula feeding with disrupted microbial diversification^28,29^, this study aims to characterize the gut microbiome of Egyptian neonates under different feeding modes.

## Materials and methods

This cross-sectional comparative study assessed how feeding mode relates to gut microbiota composition and predicted function in Egyptian infants. Forty-eight healthy full-term infants aged 0 to 6 months were recruited from pediatric outpatient clinics in Egypt during 2023 to 2024. Infants were assigned to three groups based on feeding practice, exclusive breastfeeding (N equals 12), exclusive formula feeding (N equals 18), and mixed feeding (N equals 18). Eligibility required gestational age at least 37 weeks, birth weight 2.5 to 4.0 kg, and no antibiotic probiotic or prebiotic use in the previous month. We excluded infants with congenital anomalies, prematurity, or diagnosed gastrointestinal or metabolic disease. Parents or legal guardians provided written informed consent. The Institutional Review Board at **[The Research Ethics Committee**,** Faculty of Pharmacy**,** Suez Canal University]** approved the protocol approval number **[202309MH1]** in line with the Declaration of Helsinki^30^.

### Subjects and recruitment

We used a structured questionnaire to collect infant and maternal variables including infant age and sex, maternal health history, delivery mode, and feeding practices, because these factors can influence early microbial colonization^7,11^. Recruiting healthy full-term infants reduced confounding from prematurity and clinical conditions that independently alter microbiota development^10,12^. We confirmed feeding classification using structured caregiver interviews supported by available clinical feeding records to improve group assignment accuracy^19^.

### Sample collection

We collected fresh stool samples from each infant within 24 hours after defecation using sterile DNA free polypropylene tubes. Samples were placed in insulated containers with dry ice immediately after collection and transported to the Microbiology and Molecular Biology Laboratory at Port-Said university. On arrival, we labeled samples, recorded collection and receipt times, and checked tube integrity to confirm that the cold chain was maintained. Each sample was then mixed to improve homogeneity, aliquoted into multiple sterile tubes to avoid repeated freezing thaw cycles, and stored at minus 80 degrees Celsius until DNA extraction. This workflow limits DNA degradation and reduces post collection microbial growth that can distort community profiles^24^. We followed standardized cold chain and handling steps aligned with common microbiome best practices and Human Microbiome Project style procedures to support reproducibility across samples and studies^31,32^.

### DNA extraction, quantification, PCR amplification, and sequencing

We extracted total microbial DNA from stool using the QIAamp Fast DNA Stool Mini Kit (Qiagen, Germany) and added a bead beating step to improve lysis of Gram-positive bacteria and reduce extraction bias^24^. We measured DNA quantity and purity with a NanoDrop ND 1000 spectrophotometer (Thermo Fisher Scientific, USA) and retained samples with A260 to A280 ratios between 1.8 and 2.0, then checked DNA integrity by 1% agarose gel electrophoresis. We processed extraction blanks alongside samples as negative controls to detect reagent and kit contamination^33^. For microbiome profiling, we amplified the V3 to V4 region of the bacterial 16 S rRNA gene using primers 341 F (5′ CCTACGGGNGGCWGCAG 3′) and 806R (5′ GACTACHVGGGTATCTAATCC 3′) with Illumina overhang adapters^34,35^. Each 25 µL PCR reaction contained 12.5 µL KAPA HiFi Hot Start Ready Mix, 0.2 µM of each primer, and 5 ng of template DNA, with cycling set to 95 °C for 3 min followed by 25 cycles of 95 °C for 30 s, 55 °C for 30 s, and 72 °C for 30 s, then a final extension at 72 °C for 5 min. We purified amplicons using AMPure XP magnetic beads (Beckman Coulter, USA), quantified them with a Qubit 4 fluorometer (Invitrogen, USA), pooled libraries at equimolar concentrations, and generated paired end reads (2 × 300 bp) on an Illumina MiSeq platform at IGA Technology Service (Udine, Italy) following standard Illumina 16 S workflows and QIIME2 compatible sequencing practices^36,37^.

### Sequence processing and taxonomic assignment

We processed 16 S rRNA gene sequences in QIIME 2 version 2023.2 using a standardized and reproducible workflow^37^. Raw reads were demultiplexed, quality checked, and then denoised with the DADA2 plugin to perform quality filtering, remove chimeras, and infer high resolution amplicon sequence variants rather than clustering reads into OTUs^35^. We trimmed low quality positions based on per run quality profiles and truncated reads at sites where Phred scores fell below 25 to limit sequencing error propagation into downstream diversity and taxonomy analyses. Taxonomic assignment used a pretrained Naïve Bayes classifier aligned to the SILVA rRNA reference database release 138 at 99% similarity^38^. To reduce background noise and spurious rare features, we filtered out ASVs with relative abundance below 0.01% across the dataset, consistent with common microbiome filtering practice^39^. All steps were run with recorded parameters and software versions within QIIME 2 to support full reproducibility^35^.

### Statistical and diversity analysis

We quantified within sample diversity using Observed Species and Chao1 to capture richness, and Shannon and Simpson indices to reflect both richness and evenness across taxa^40,41^. We compared alpha diversity across feeding groups with the Kruskal Wallis test. We measured sample differences using Bray Curtis dissimilarity and weighted UniFrac distances to capture both abundance-based variation and phylogenetic relatedness^41,42^. We tested beta diversity separation among groups using PERMANOVA and considered p below 0.05 as significant^43^. To control for potential confounders, we performed PERMANOVA (function adonis2 in R package vegan version 2.6-4) with feeding mode as the main predictor and infant age (days), delivery mode (vaginal/C-section), and sex (male/female) as covariates. Significance was assessed using 999 permutations. We also calculated the Firmicutes to Bacteroidetes ratio from relative abundances as an ecological indicator linked to community level balance in early life studies^44^. To identify taxa associated with each feeding mode, we applied LEfSe and reported features with an LDA score above 2.0 at alpha 0.05, which supports interpretable group specific microbial signatures^45^. We generated figures and ordination plots in R version 4.3.0 using phyloseq microbiome and ggplot2 to ensure consistent processing and reproducible visualization^46^.A post-hoc power analysis was performed using G*Power software (version 3.1.9.7) based on the observed effect sizes for the Shannon diversity index (Cohen’s f = 0.45). The achieved power was 0.76, indicating that the study was adequately powered to detect large effect sizes. However, we acknowledge the imbalance in group sizes (12 exclusively breastfed vs. 18 in each of the other groups) as a limitation of this exploratory study. For stacked phylum-level bar plots, error bars were omitted due to compositional constraints (relative abundances sum to 100% per sample). For genus-level bar plots, 95% confidence intervals are shown as a measure of precision around the mean.

### Functional prediction and correlation analysis

We inferred community functional potential from 16S profiles using PICRUSt2 to predict KEGG ortholog content and pathway representation^47,48^, focusing on pathways relevant to early life biology including carbohydrate utilization, amino acid biosynthesis, vitamin related metabolism, and host immune interaction functions^49^. To predict the functional potential of the microbial communities, we used PICRUSt2 (version 2.5.2)^49^, which first placed the ASV representative sequences into a reference phylogeny using SEPP, then inferred abundances of KEGG Orthologs (KOs) via hidden Markov model searches against the Integrated Microbial Genomes (IMG) database^47,48^; predicted KO counts were normalised by copy number and summed across ASVs per sample to generate a KO abundance table, which was subsequently collapsed to MetaCyc pathway abundances using the MinPath algorithm. Differential abundance of KOs and pathways between feeding groups (breastfed, artificially fed, mixed fed) was assessed using two complementary methods: DESeq2 (version 1.40.0) with a negative binomial generalised linear model and Wald test, applying Benjamini‑Hochberg correction for multiple testing (padj < 0.05), and LEfSe with a Kruskal‑Wallis significance threshold of *p* < 0.05 and an LDA score cut‑off of 2.0. Only KOs that were significant by both DESeq2 and LEfSe were considered robust functional biomarkers of feeding mode. We then tested associations between microbial features and key variables such as feeding mode, delivery type, and infant age using Spearman rank correlations, and we controlled for multiple testing using false discovery rate correction to reduce false positive findings common in high dimensional microbiome data^50^. We produced heatmaps, ordination plots, and correlation-based network diagrams in R to summarize patterns and highlight the strongest relationships. For correlation networks (Figs. 5 and 6), we included only Spearman correlations with |*r*| > 0.5 and FDR-corrected *p*-value < 0.05. Laboratory handling followed sterile, DNA free procedures throughout to limit contamination risk, and all computational steps followed reproducible bioinformatics practice with documented software versions and parameters^32,37^.

## Results

### Phylum level microbiome composition across infant feeding groups

**Fig. 1:**
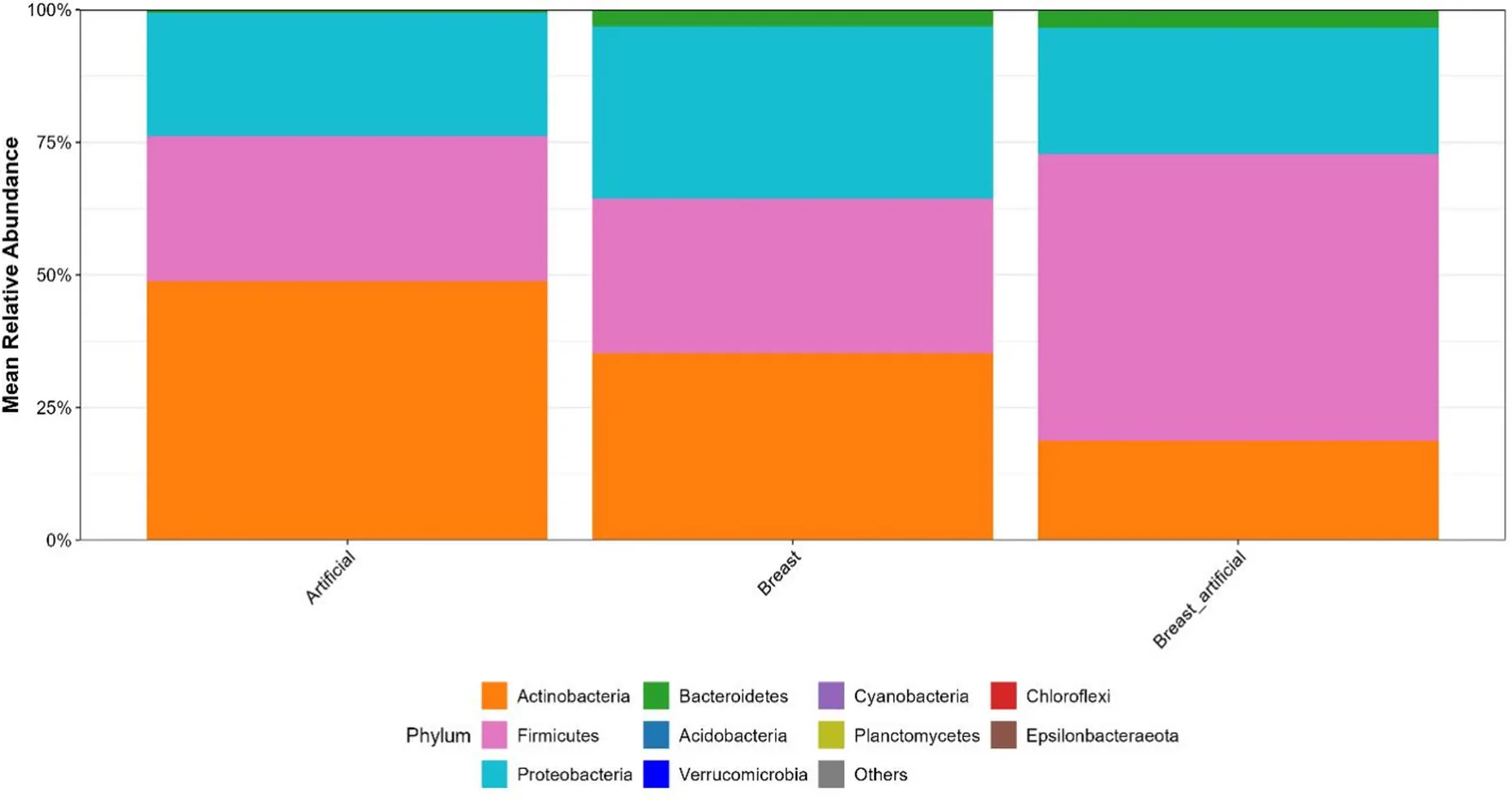
Phylum-level composition of the infant gut microbiota across feeding groups.

Stacked bar plots show the mean relative abundance of bacterial phyla in artificially fed, exclusively breastfed, and mixed-fed infants. Each bar represents the average proportional contribution of major phyla, including *Actinobacteria*,* Firmicutes*,* Proteobacteria*,* Bacteroidetes*, and lower-abundance phyla grouped as others. Differences in phylum-level profiles illustrate feeding-associated shifts in community structure, with variation in the relative dominance of major bacterial phyla across feeding types.

In this report, 48 infant fecal samples were successfully sequenced. After quality filtering and denoising, a median of about 45,000 high quality reads per sample was retained. Taxonomy was assigned using the SILVA 138 reference database, yielding over 300 ASVs across 10 bacterial phyla, 25 families, and 57 genera across all samples. Richness was similar across feeding groups, while relative abundances shifted substantially between groups. The mean (± *SD*) age in days was: exclusively breastfed group 89.4 (± 45.2), formula-fed group 95.1 (± 41.6), and mixed-fed group 92.7 (± 43.8). The median (range) age was: breastfed 78 (15–180), formula-fed 85 (18–182), and mixed-fed 84 (14–178). At the phylum level, *Actinobacteria*,* Firmicutes*, and *Proteobacteria* accounted for most of the relative abundance in each feeding group. *Bacteroidetes* showed low relative abundance in the breastfed and mixed groups and was nearly absent in the artificial feeding group. The remaining phyla each contributed only a small fraction of the community at the scale shown and therefore do not form distinct layers in the group mean stacked bar plot **(**Fig. 1**).**

Artificial feeding produced the most *Actinobacteria*-heavy profile. *Actinobacteria* accounted for 48.5% of mean relative abundance, *Firmicutes* for 28.0%, and *Proteobacteria* for 23.5%, with no visible *Bacteroidetes* layer in this group. Breastfeeding shifted that balance. Breastfed infants showed *Actinobacteria* at 34.8%, *Firmicutes* at 29.4%, *Proteobacteria* at 32.8%, and a small *Bacteroidetes* contribution at 2.9%. Compared with artificial feeding, breastfeeding reduced *Actinobacteria* by about 13.7% points and increased *Proteobacteria* by about 9.3% points, while Firmicutes stayed close and Bacteroidetes appeared as a consistent low-abundance component **(**Fig. 1**).** Mixed feeding produced the most distinct phylum profile in the figure because *Firmicutes* became dominant. In the mixed feeding group, *Firmicutes* reached 54.8% of mean relative abundance, while *Proteobacteria* accounted for 24.3%, *Actinobacteria* for 18.0%, and *Bacteroidetes* for 2.9%. Relative to breastfeeding alone, mixed feeding increased *Firmicutes* by about 25.4% points and reduced *Actinobacteria* by about 16.8% points, while *Proteobacteria* decreased by about 8.5% points and *Bacteroidetes* stayed similar **(**Fig. 1**).** Relative to artificial feeding alone, mixed feeding reduced *Actinobacteria* by about 30.5% points and increased *Firmicutes* by about 26.8% points, with *Proteobacteria* remaining in the same mid-range and *Bacteroidetes* staying low.

### Alpha diversity differed across feeding groups, delivery modes and sex

**Fig. 2:**
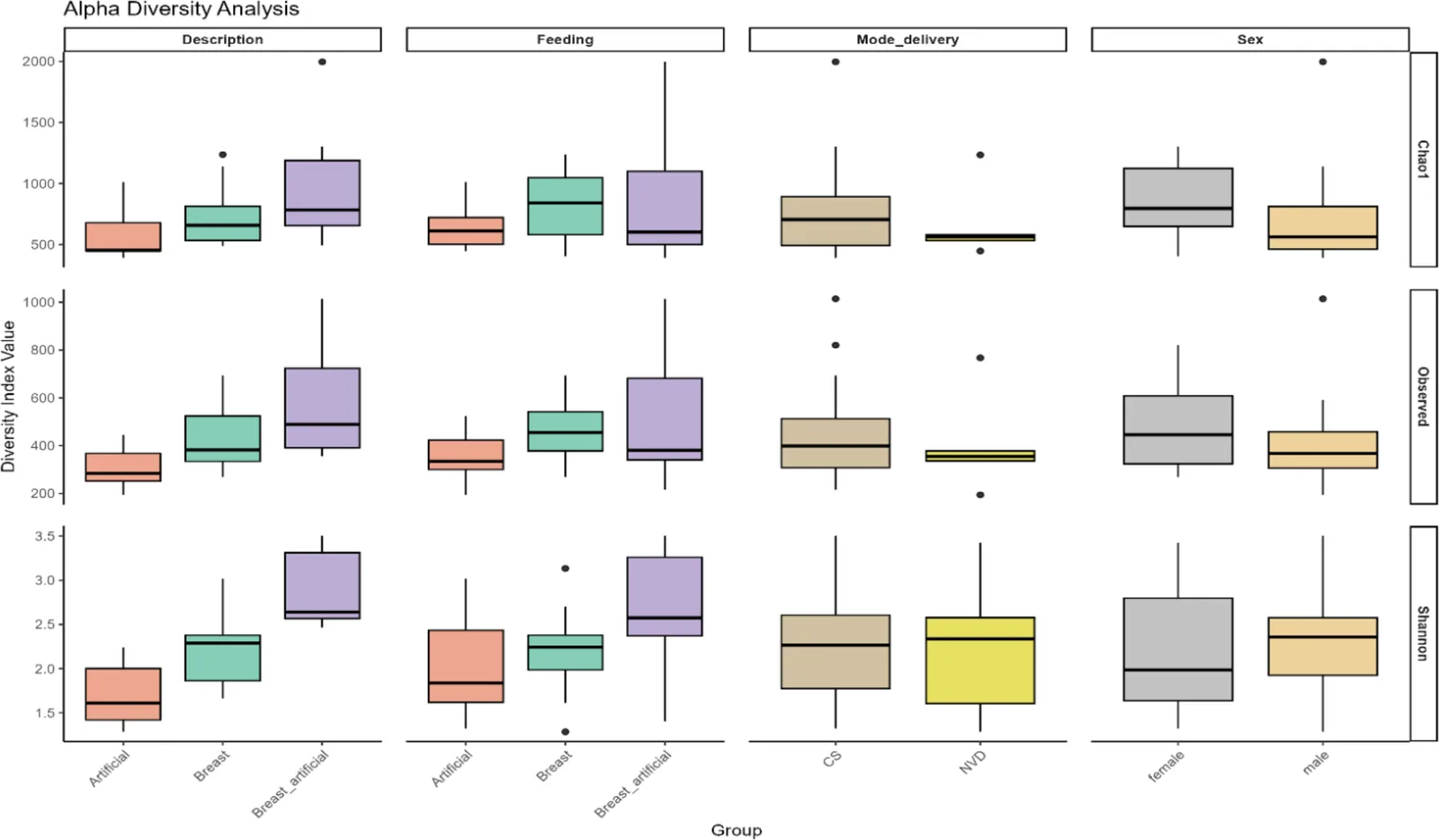
Alpha diversity of the infant gut microbiota across clinical and demographic variables.

Alpha diversity indices including Chao1, observed richness, and Shannon diversity are shown across feeding type, mode of delivery, and infant sex. Boxplots display the distribution of diversity values within each group, with the median indicated by the central line, interquartile range by the box, and whiskers representing data spread; points indicate outliers. Feeding groups include artificial feeding, exclusive breastfeeding, and mixed feeding. Mode of delivery includes cesarean section and normal vaginal delivery. Sex includes female and male infants. This figure illustrates how feeding type is associated with differences in microbial richness and diversity relative to delivery mode and sex. Beta diversity assessed by Principal Coordinate Analysis.

**Fig. 3:**
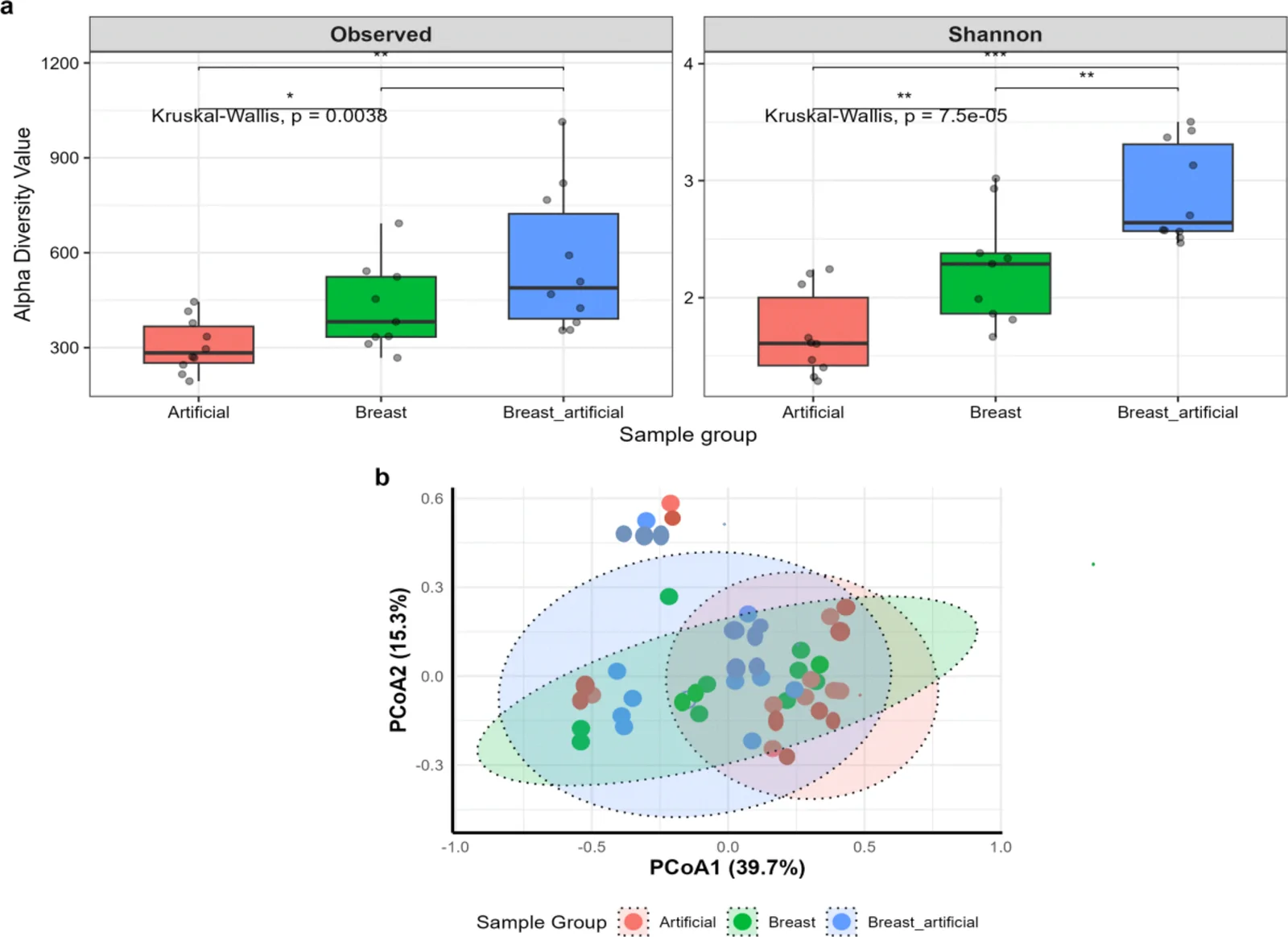
Alpha and beta diversity of the infant gut microbiota across feeding groups. (**a**) Alpha diversity measured by Observed richness and Shannon diversity index in artificially fed, exclusively breastfed, and mixed-fed infants. Boxplots show median and interquartile range, with individual points representing samples. Group differences were assessed using the Kruskal–Wallis test, and pairwise comparisons are indicated by significance bars. (**b**) Principal coordinates analysis (PCoA) based on community dissimilarity, showing separation of samples by feeding group. Each point represents one sample, colors indicate feeding groups, and shaded ellipses denote group dispersion. The percentage of variance explained by each axis is shown on the axes.

In this report, an association between feeding mode and microbial richness and evenness was announced, which was confirmed using the alpha diversity based on Chao1, Observed index and Shannon index across all feeding groups. Chao1 richness varied across feeding groups **(**Fig. 2**).** Artificial feeding showed the lowest median Chao1 values. Breastfeeding showed higher value. Mixed feeding showed the highest median and the widest spread. For the Observed index, artificial feeding showed the lowest median values. Breastfeeding showed higher value. Mixed feeding showed the highest values. The Kruskal Wallis test indicated a significant overall difference p 0.0038. Pairwise comparisons showed differences between artificial feeding and breastfeeding and a stronger difference between artificial feeding and mixed feeding. A difference was also present between breastfeeding and mixed feeding as indicated in the plot. For the Shannon index, artificial feeding again showed the lowest diversity. Breastfeeding showed higher diversity. Mixed feeding showed the highest diversity. The Kruskal Wallis test showed a strong overall effect p 7.5e minus 5. Pairwise comparisons indicated significant differences between artificial and breastfeeding, artificial and mixed feeding, and between breastfeeding and mixed feeding. Consistent with these patterns, the Kruskal–Wallis testing **(**Fig. 3**)** supported global differences across feeding groups for both richness and diversity, with significant effects for the Observed index (*p* = 0.0038) and the Shannon index (*p* = 7.5 × 10⁻⁵). These patterns indicated greater estimated taxonomic richness in infants receiving breast milk, particularly when combined with artificial feeding. Within group variability was substantial, especially in the mixed feeding group, indicating heterogeneous richness across individuals. Therefore, there was a stepwise increase within sample diversity from artificial feeding to breastfeeding to mixed feeding.

Alpha diversity stratified by delivery mode is shown in (Fig. 2**)**. Across Chao1, Observed, and Shannon, the distributions for cesarean section and vaginal delivery overlap strongly. Median differences appear modest relative to the within group spread. Any delivery mode signal is more apparent for richness focused indices Chao1 and Observed than for Shannon, which suggests that delivery mode relates mainly to the number of taxa detected rather than to community evenness. Alpha diversity stratified by infant sex is shown in (Fig. 2**)**. Chao1, Observed, and Shannon values are similar between females and males. The medians are close and the interquartile ranges overlap across all three indices. This indicates no clear sex associated pattern in richness or diversity in this analysis. Therefore, the alpha diversity patterns by feeding are stronger than those by delivery mode or sex. Delivery mode and sex show overlapping distributions with modest shifts relative to feeding. This indicates that feeding explains more variation in alpha diversity in this dataset than delivery mode or sex.

### Feeding group differences in infant gut microbiota beta diversity and community dispersion

Principal Coordinate Analysis based on beta diversity distances was used to compare gut microbial community structure across different feeding groups **(**Fig. 3B**).** The breastfeeding group showed a shift in location relative to the artificial feeding group along PCoA1, while mixed feeding samples occupied an intermediate position and overlapped with both groups. The breastfeeding group showed a more compact distribution in the ordination space, indicating a relatively homogeneous gut microbiota among breastfed infants. In contrast, the artificial feeding group exhibited a broader dispersion, reflecting greater variability in microbial community composition. The mixed feeding group occupied an intermediate position and overlapped with both breastfeeding and artificial feeding groups, consistent with a transitional community structure that shared features of both feeding modes. The confidence ellipses overlapped substantially across all three groups, and points from each feeding group occurred within the overlap region. PERMANOVA supported the ordination patterns. Community structure differed between breastfeeding and artificial feeding (*R²* = 0.142, *p* = 0.001) and between breastfeeding and mixed feeding (*R²* = 0.103, *p* = 0.001), indicating that feeding group explained a measurable but limited proportion of variation in beta diversity. In contrast, artificial feeding and mixed feeding did not differ significantly (*R²* = 0.042, *p* = 0.083), consistent with their overlap in the ordination. Notably, PERMANOVA controlling for age, delivery mode, and sex confirmed that feeding mode remained a significant driver of beta diversity (*R²* = 0.098, *p* = 0.014) after accounting for these confounders. Therefore, beta diversity results indicated that breastfeeding was associated with a shift in gut microbial community structure relative to artificial and mixed feeding, while artificial and mixed feeding showed more similar community profiles. The overlap across groups suggested that factors beyond feeding contributed to inter individual differences in gut microbiome composition.

### Firmicutes bacteroidetes ratio dynamics and genus level differential abundance

**Fig. 4:**
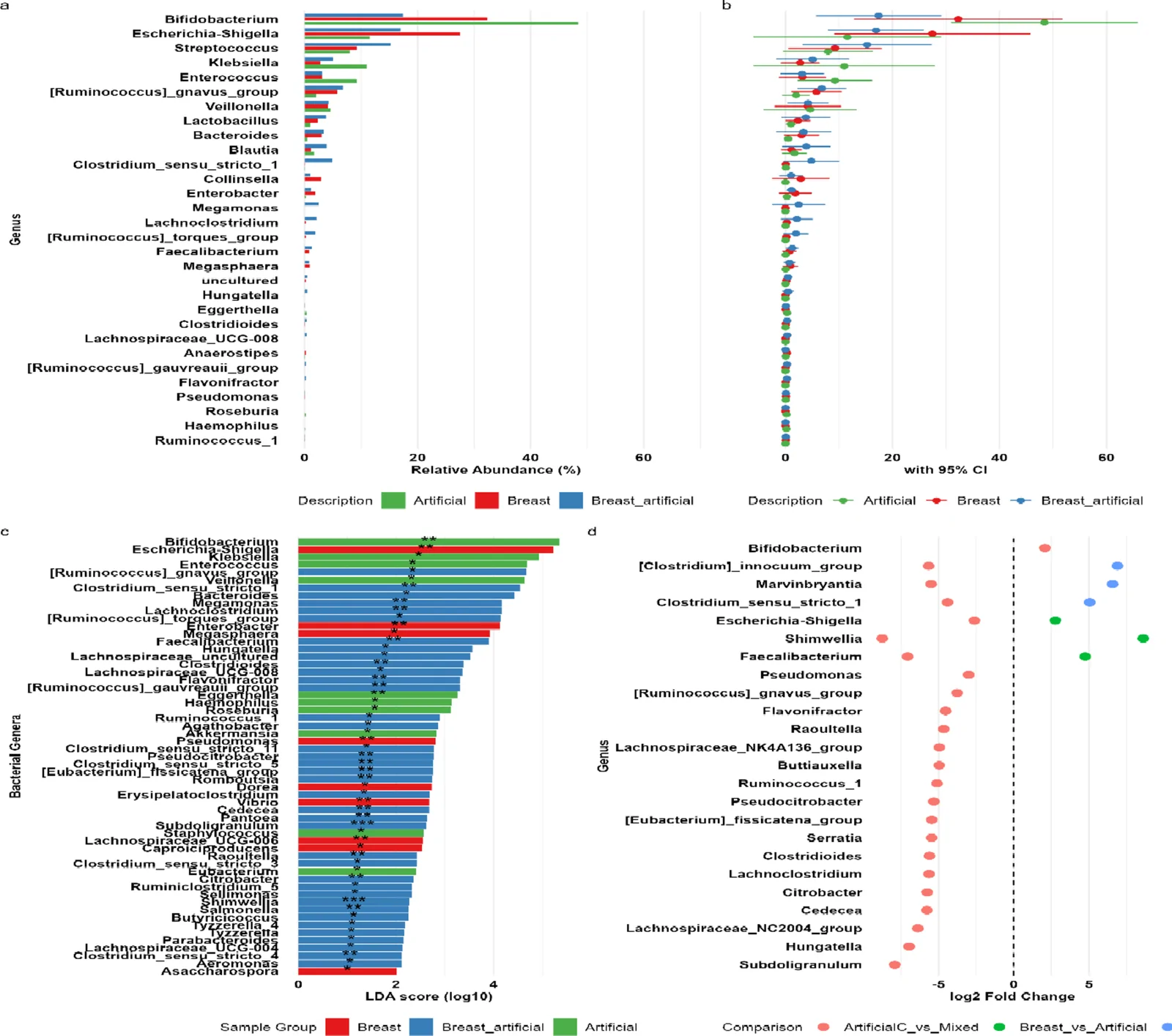
Feeding-associated differences in the infant gut microbiota at the genus level. (**a**) Relative abundance of the most prevalent bacterial genera across feeding groups including artificial feeding, exclusive breastfeeding, and mixed feeding. Genera are ordered by overall abundance, and bars represent mean relative abundance within each group. (**b**) Mean relative abundance of the same genera with 95% confidence intervals, highlighting between-group differences for dominant taxa and overlap among lower-abundance genera. (**c**) Linear Discriminant Analysis Effect Size (LEfSe) results identifying discriminative bacterial genera associated with each feeding group; bars indicate LDA scores (log10) reflecting the magnitude of group-specific enrichment. (**d**) Pairwise differential abundance analysis showing log2 fold changes in genus abundance between feeding groups, with points colored by comparison and the dashed vertical line indicating no change. Positive values indicate enrichment in the reference group, and negative values indicate depletion.

The *Firmicutes Bacteroidetes* (F/B) ratio differed across feeding groups and aligned with shifts in dominant genera. In the artificial feeding group *Bacteroidetes* were nearly absent which made the F/B ratio effectively unbounded and indicated strong phylum level imbalance. Exclusively breastfed infants showed *Firmicutes* near 29% and *Bacteroidetes* near 3% which yields an F/B ratio near 9.67 and indicated lower *Firmicutes* dominance than the formula exposed groups. Mixed fed infants showed *Firmicutes* near 54% with *Bacteroidetes* near 3% which yields an F/B ratio near 18 and indicates the strongest Firmicutes skew among groups with detectable *Bacteroidetes*. At the genus level the relative abundance profiles in Fig. 4A showed that *Bifidobacterium* dominated all groups and reached the highest abundance in the artificial feeding group while breastfed infants showed higher *Escherichia*, *Shigella* and mixed fed infants showed higher *Streptococcus* and *Klebsiella*. Group means and 95% confidence intervals in Fig. 4B confirmed the same dominant genus differences and showed broad overlap for most low abundance genera which indicates that a small set of dominant taxa explains most between group separation.

Genus level biomarker and differential abundance analyses showed distinct microbial signatures across feeding modes. Linear Discriminant Analysis Effect Size (LEfSe) in Fig. 4C identified 21 discriminative taxa using Linear Discriminant Analysis (LDA) greater than 2 and, *p-value* below 0.05. Artificial feeding was characterized primarily by *Bifidobacterium* as the strongest biomarker LDA 5.35, *p-value* 0.0467. Breastfeeding was characterized by enrichment of *Escherichia-Shigella* LDA 4.85, *p-value* 0.022 together with *Enterobacter* LDA 4.17, *p-value* 0.031, *Pseudomonas* LDA 3.64, *p-value* 0.042, and *Vibrio* LDA 3.33, *p-value* 0.047 which defined a breastfeeding associated signature dominated by facultative anaerobes. Mixed feeding showed enrichment of *Streptococcus* alongside several *clostridial* and *Enterobacteriaceae* related genera, including *Subdoligranulum* LDA 4.67, *p-value* 0.029, Eubacterium *fissicatena* group LDA 4.52, *p-value* 0.037, *Clostridioides* LDA 3.94, *p-value* 0.048, *Citrobacter* LDA 4.38, *p-value* 0.031, and *Raoultella* LDA 4.12, *p-value* 0.041, consistent with a mixed profile that combined fermentative SCFA associated taxa with genera that can include opportunistic members.

Pairwise differential abundance in Fig. 4D supported these shifts, with breastfeeding versus artificial feeding showing enrichment of *Escherichia-Shigella* (log_2_FC = 2.77, *p-value* = 0.0042), enrichment of *Faecalibacterium* (log_2_FC = 4.75, *p-value =* 0.032), and enrichment of *Shimwellia* (log_2_FC = 8.61, *p-value* < 0.001) in breastfed infants. Artificial feeding versus mixed feeding in Fig. 4D showed depletion in artificial feeding of short-chain fatty acid (SCFA) associated genera including *Faecalibacterium* log_2_FC= -7.06, *p-value =* 2.2 × 10^− 4^), *Subdoligranulum* (log_2_FC = − 7.91, *p-value =* 0.029), and *Shimwellia* (log_2_FC= -8.75, *p-value* < 0.001) with additional reductions in clostridial groups including *Clostridium sensu stricto 1* and *Lachnoclostridium* and only modest non-significant enrichment of *Bifidobacterium*. Integrated with alpha diversity these findings indicate that artificial feeding combines extreme phylum imbalance with lower richness and evenness and reduced representation of key fermentative genera; while breastfeeding and mixed feeding show higher alpha diversity and retain taxa linked to fermentation and immune relevant metabolites, with mixed feeding showing an intermediate pattern combining features of breastfeeding and formula exposure.

### Genus-level differential abundance and top 20 genera across feeding groups

At the genus level, microbial composition differed between feeding groups. The top 20 genera accounted for more than 90% of total relative abundance and varied by feeding type **(**Table 1**).** These patterns indicate that feeding mode modulates the gut microbiota of Egyptian neonates, with breastfeeding associated with an early colonization profile dominated by facultative anaerobes and formula feeding associated with a more specialized community structure. In breastfed infants, the dominant genera were *Escherichia-Shigella* 18.2%, *Enterobacter* 12.6%, *Pseudomonas* 8.9%, and *Vibrio* 6.3%, consistent with early colonizers that can reduce luminal oxygen and support gut maturation. In formula fed infants, *Bifidobacterium* 21.5% was the dominant genus, followed by *Streptococcus* 10.4% and *Veillonella* 7.2%, which is consistent with adaptation to formula associated carbohydrate substrates and a lower overall diversity. Mixed fed infants showed a transitional profile, with *Subdoligranulum* 12.4%, *Eubacterium fissicatena* group 9.6%, *Citrobacter* 8.1%, and *Raoultella* 5.8%, indicating a community that includes butyrate associated fermenters alongside genera that can include opportunistic members.

**Table 1:** The top 20 genera accounted for more than 90% of total relative abundance and varied according to feeding type.

Group	Dominant Genera (mean % ± SD)	Key Notes
Breastfed	*Escherichia-Shigella (18.2 ± 5.1)*,* Enterobacter (12.6 ± 4.3)*	Early colonizers; oxygen-reducing facultative anaerobes supporting gut maturation
Formula-fed	*Bifidobacterium (21.5 ± 6.4)*,* Streptococcus (10.4 ± 3.9)*	Reflect adaptation to formula carbohydrates; lower overall diversity.
Mixed-fed	*Subdoligranulum (12.4 ± 4.1)*,* Eubacterium fissicatena group (9.6 ± 3.4)*	Transitional profile including both beneficial butyrate producers and opportunistic taxa.

Pairwise comparisons (Kruskal-Wallis with Dunn’s post-hoc test) showed significant differences for Escherichia-Shigella between breastfed and formula-fed (*p* = 0.004) and between breastfed and mixed-fed (*p* = 0.021).

### Functional prediction of gut microbiota (PICRUSt2 analysis)

Functional profiling based on KEGG Ortholog (KO) predictions revealed distinct metabolic signatures associated with infant feeding modes. The breastfed group was characterized by enrichment of *Bifidobacterium*-associated KOs involved in human milk oligosaccharide (HMO) degradation (K01190, log₂FC = 4.2, *p =* 0.001, LDA = 4.5) and oligosaccharide fermentation (K01187, log₂FC = 3.9, *p =* 0.002, LDA = 4.3), alongside *Lactobacillus*-mediated lactate-to-acetate conversion (K01034, log₂FC = 3.5, *p =* 0.003, LDA = 4.1). Additional saccharolytic and butyrogenic pathways, including acetate production by *Blautia* (K00925, log₂FC = 2.8, *p =* 0.006, LDA = 3.6), butyrate synthesis by *Faecalibacterium* (K01679, log₂FC = 2.6, *p =* 0.008, LDA = 3.4), and mucin glycan degradation by *[Ruminococcus] gnavus* (K01006, log₂FC = 2.4, *p =* 0.01, LDA = 3.2), were also enriched in breastfed infants.

In contrast, the artificial feeding group exhibited a pro-inflammatory and oxidative stress-prone profile, with significant enrichment of *Enterococcus* lactate production (K00016, log₂FC = 4.5, *p =* 0.0008, LDA = 4.7), *Escherichia-Shigella* LPS biosynthesis (K00656, log₂FC = 4.1, *p =* 0.001, LDA = 4.4), and multiple *Streptococcus*-associated oxidative stress enzymes (glutathione peroxidase K00383, LDA = 3.8; glutathione reductase K00382, LDA = 3.7; superoxide dismutase K04564, LDA = 3.6). *Klebsiella* motility (flagellar proteins K02389, K02392, LDA ~ 3.4–3.5) and nitrate reduction (K02003, LDA = 3.7) were also enriched, alongside *Clostridium sensu stricto 1* mixed acid fermentation (K00925, K00276, LDA = 3.8–3.9).

The mixed feeding group displayed a hybrid functional landscape, featuring methanogenesis (*Methanobrevibacter* K00400, LDA = 4.6), butyrate production (multiple *Faecalibacterium*,* Roseburia*,* Lachnospiraceae*,* Agathobacter*,* Subdoligranulum*,* Coprococcus* with LDA 2.4–4.4), cholesterol conversion (*Eubacterium* K00783, LDA = 4.1), bile salt hydrolase activity (*Parabacteroides* K15872, LDA = 3.0), and polysaccharide degradation (*Prevotella 9*,* Barnesiella*). Notably, *Veillonella* and *Campylobacter* oxidative stress/motility genes appeared in both artificial and mixed feeding groups, suggesting potential overlap in inflammatory or dysbiotic signatures (Table 2).

**Table 2:** Differentially abundant KEGG Orthologs (KOs) predicted by PICRUSt2 across feeding groups (LEfSe analysis).

Feeding Group	Genus	LDA Score	*p*-value	Key Predicted Function
**Breastfeeding**	*Bifidobacterium*	4.3–4.5	0.0005–0.001	HMO degradation, oligosaccharide fermentation
	*Lactobacillus*	4.1	0.002	Lactate→acetate conversion
	*Blautia*	3.6	0.004	Acetate production
	*Faecalibacterium*	3.4	0.005	Butyrate synthesis
	*[Ruminococcus] gnavus*	3.2	0.007	Mucin glycan degradation
	*Bacteroides*	2.9	0.01	Proteolytic fermentation
**Artificial feeding**	*Enterococcus*	4.7	0.0004	Lactate production (pro-inflammatory)
	*Escherichia-Shigella*	4.4	0.0007	LPS biosynthesis
	*Streptococcus*	3.6–3.8	0.001–0.002	Oxidative stress enzymes (GPx, GR, SOD)
	*Klebsiella*	3.3–3.7	0.002–0.004	Motility, nitrate reduction
	*Enterobacter*	3.3	0.005	Opportunistic pathogen signature
	*Clostridium s.s. 1*	3.8–3.9	0.002–0.003	Mixed acid fermentation
	*Veillonella*	3.1–3.7	0.002–0.008	Iron transport, oxidative stress
**Mixed feeding**	*Methanobrevibacter*	4.6	0.0005	Methanogenesis (H₂ utilization)
	*Prevotella_9*	4.5	0.0006	Polysaccharide degradation
	*Phascolarctobacterium*	4.2	0.001	Succinate→propionate
	*Eubacterium*	4.1	0.001	Cholesterol→coprostanol
	*Lachnospiraceae NK4A136*	4.0	0.002	Butyrate production
	*Agathobacter*	3.8	0.003	Butyrate production
	*Collinsella*	3.7	0.003	Hydrogen-producing fermentation
	*Megasphaera*	3.5	0.004	Succinate→propionate
	*Barnesiella*	3.4	0.005	Polysaccharide degradation
	*Parabacteroides*	3.0	0.008	Bile salt hydrolase (BSH)
	*Roseburia*	2.8	0.01	Butyrate production
	*Subdoligranulum*	3.2	0.007	Butyrate production
	*Coprococcus*	2.4	0.03	Butyrate production

### Correlation analysis highlights feeding-specific microbial associations

**Fig. 5:**
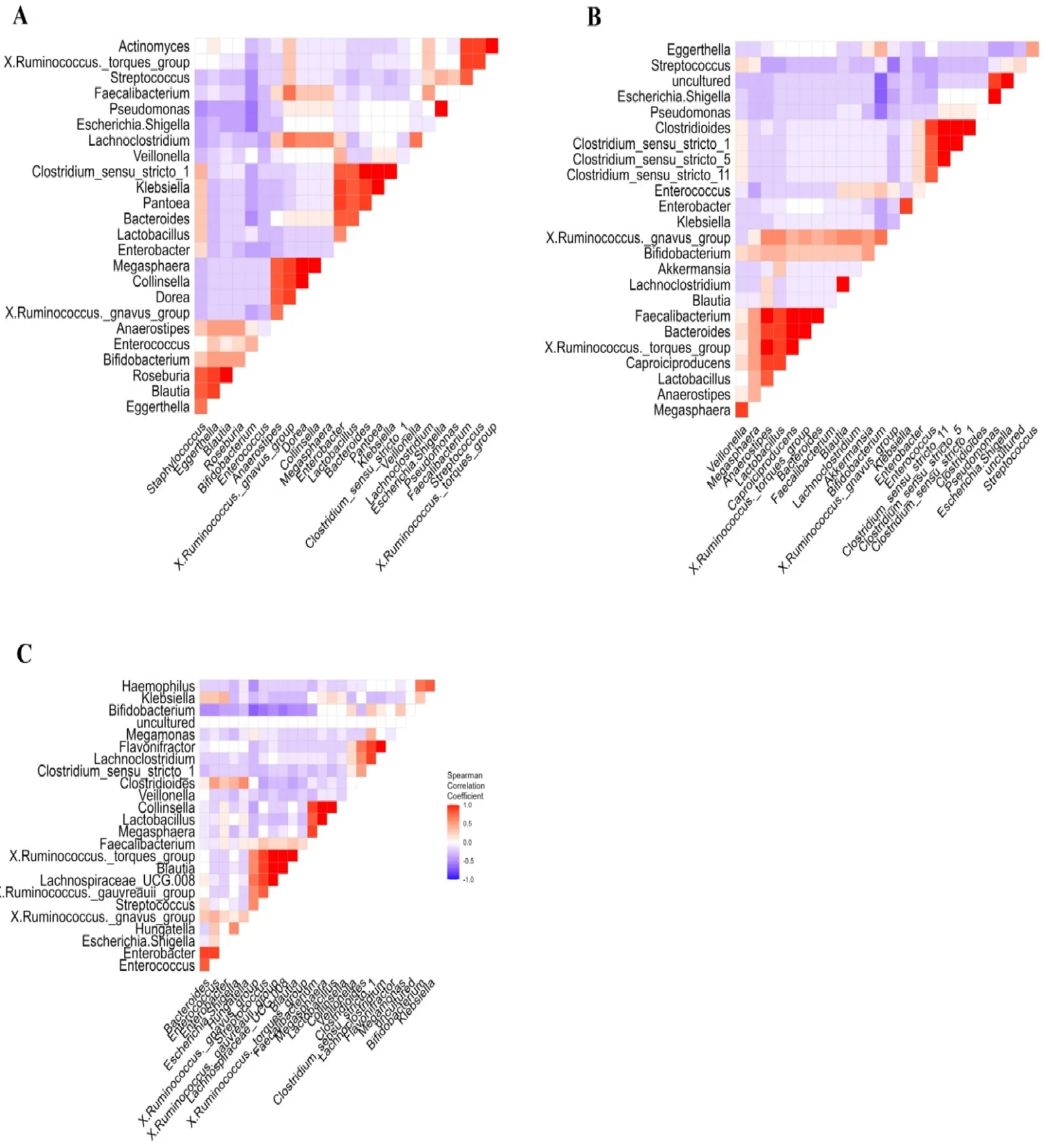
Spearman correlation heatmaps for three datasets. The figure displays pairwise Spearman correlation coefficients among scaled variables for (**A**) Artificial dataset, (**B**) Breast dataset, and (**C**) Mixed dataset. Hierarchical clustering was applied to group variables with similar correlation patterns. Blue indicates negative correlations, red indicates positive correlations, and white represents near-zero correlations. Only correlations with *|r|* > 0.5 and FDR-corrected *p* < 0.05 are shown.

Comparative analysis of the correlation heatmap in panel A showed structured co-occurrence patterns among dominant genera in the artificial feeding group (Fig. 5A**)**. A block of opportunistic taxa including *Klebsiella*,* Bacteroides*,* Enterobacter*, and *Clostridium _sensu_stricto_1* exhibited strong positive correlations, with R-values largely in the 0.7 to 0.9 range based on the red intensity of the heatmap (Fig. 5A**)**. Within this block, *Clostridium_sensu_stricto_1* showed particularly strong associations with *Klebsiella* and *Bacteroides*, approaching the upper end of the scale. *Streptococcus* showed a positive association with *Actinomyces* with R-values around 0.6 to 0.7, while its relationship with *Faecalibacterium* appeared weak to moderate and did not exceed mid-range correlation strength. *Bifidobacterium* displayed negative correlations with *Proteobacteria* such as *Escherichia*. *Shigella* and *Pseudomonas*, with inferred R-values in the mild to moderate negative range of approximately − 0.3 to − 0.5. Overall, the figure supported strong positive co-occurrence among opportunistic genera and weaker antagonistic relationships involving *Bifidobacterium*, with correlation strength best interpreted as approximate ranges rather than exact values.

The correlation heatmap that indicated organized co-occurrence patterns among the dominant genera in the breastfed group was presented In (Fig. 5B**)**. A strongly connected cluster composed of anaerobic commensals such as *Faecalibacterium*, *Blautia*, *Bacteroides*, *X.Ruminococcus_torques_*group, and *Caproiciproducens* displayed high positive correlations, with *R*-values mostly around 0.7 to 0.9 as suggested by the deep red signals in (Fig. 5B**)**. Within this cluster, *Faecalibacterium* showed the strongest links, particularly with *Blautia* and *Bacteroides*, with values near the top of the scale. *Bifidobacterium* was positively associated with *Akkermansia* and *X.Ruminococcus_gnavus*_group at a moderate level, with *R-values* approximately 0.4 to 0.6. By contrast, *Proteobacteria* including *Escherichia.Shigella*,* Pseudomonas*,* and Klebsiella* showed weak to negative relationships with multiple commensal taxa, generally falling near *r* = − 0.2 to − 0.4. Collectively, (Fig. 5B**)** supported a breastfed associated network dominated by strong positive co-occurrence among anaerobic commensals and comparatively reduced connectivity involving *Proteobacteria*, with correlation magnitudes best treated as approximate ranges rather than exact coefficients.

Comparative analysis of the correlation heatmap in (Fig. 5C**)** showed a mixed and transitional co-occurrence structure among dominant genera in the mixed feeding group. A core cluster of anaerobic commensals including *Faecalibacterium*, *Blautia*, X.*Ruminococcus*_ *torques_group*, *Lachnospiraceae*_UCG_008, and X.*Ruminococcus*_ *gauvreauii*_group exhibited moderate to strong positive correlations, with inferred r values largely in the range of approximately 0.6 to 0.8 based on the red intensity of the heatmap. Within this cluster, *Faecalibacterium* showed particularly strong associations with *Blautia* and X.*Ruminococcus*_ *torques_group*. *Lactobacillus* and *Megasphaera* also demonstrated positive correlations with several anaerobic taxa, although these associations appeared weaker and more variable. In contrast, *Proteobacteria* such as *Enterobacter*,* Escherichia. Shigella*,* Klebsiella*, and *Haemophilus* showed weak to negative correlations with many commensal genera, with inferred r values generally ranging from − 0.2 to − 0.5. *Bifidobacterium* displayed heterogeneous associations, including weak negative correlations with Proteobacteria and limited positive connectivity within the anaerobic cluster. Overall, (Fig. 5C**)** supported a mixed feeding associated network characterized by intermediate connectivity patterns that shared features of both breastfed and artificially fed microbial communities, with correlation strengths best interpreted as approximate ranges rather than exact coefficients.

### Distinct microbial co variation networks associated with infant feeding practices

**Fig. 6:**
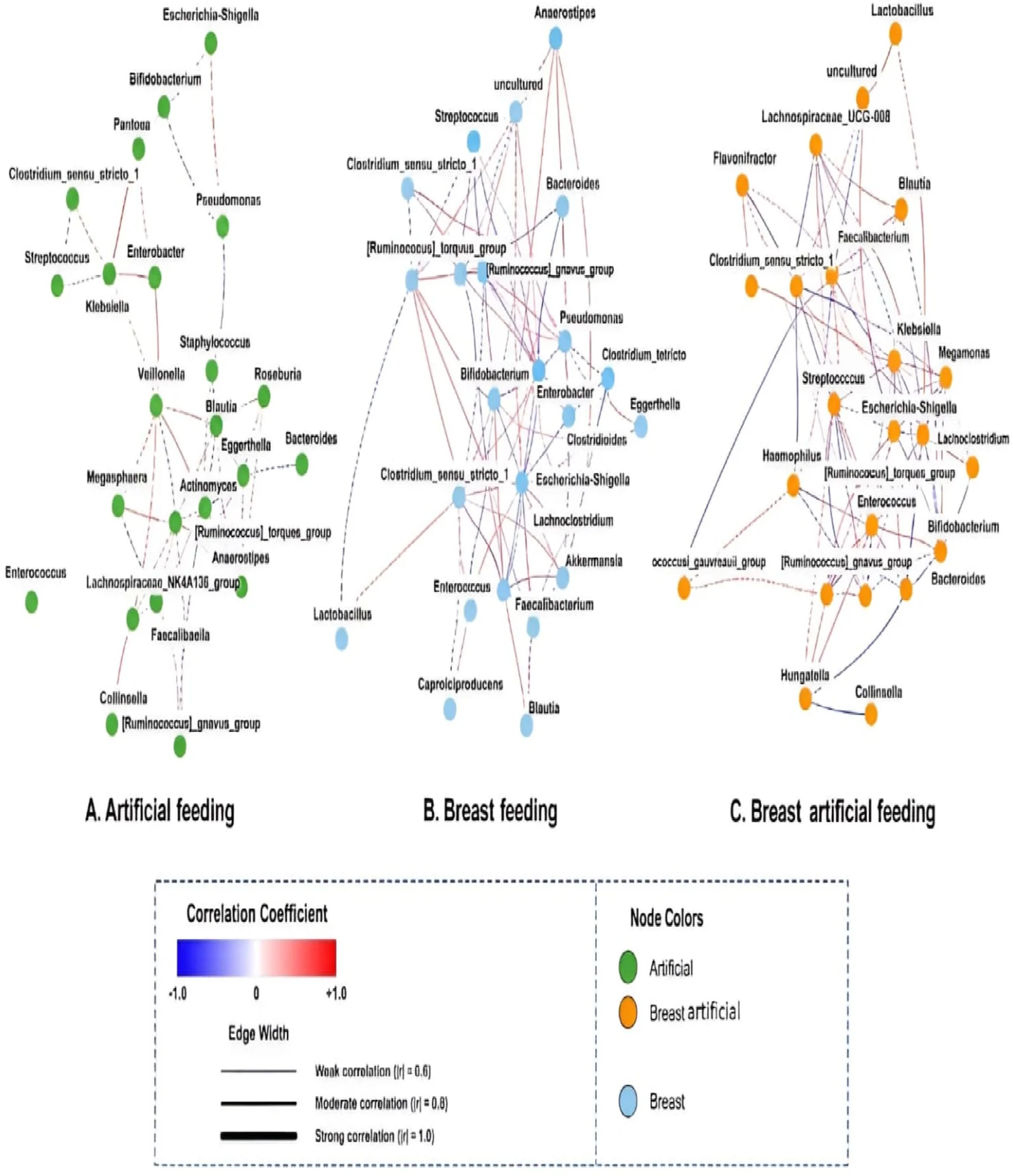
Correlation networks of infant gut microbiota across feeding types. Genus-level correlation networks were constructed separately for (**A**) artificial, (**B**)breast, and (**C**) mixed-fed infant groups. Nodes represent bacterial genera, and edges represent pairwise correlations in relative abundance across samples within each feeding group. Edge color indicates correlation direction, with red representing positive correlations and blue representing negative correlations, while edge width reflects correlation strength, with thicker edges indicating stronger absolute correlation values. Overall, the figure illustrates feeding-type–specific differences in network topology, connectivity, and patterns of co-occurrence and co-exclusion among dominant gut bacterial genera. Only correlations with *|r|* > 0.5 and FDR-corrected *p* < 0.05 are shown.

The artificial feeding network showed a moderately connected and organized structure with several central genera **(**Fig. 6A**)** including *Escherichia–Shigella*,* Enterobacter*,* Streptococcus*,* Veillonella*,* and Bifidobacterium*, each linked to multiple taxa, indicating structured co-variation patterns across samples. Both positive and negative correlations were present, reflecting cooccurrence and co-exclusion relationships rather than direct ecological interactions. Anaerobic and fermentative genera including *Faecalibacterium*,* Blautia*,* Dorea*, and members of *Lachnospiraceae* formed a connected subcluster within the network, suggesting coordinated variation among taxa commonly associated with carbohydrate fermentation. Overall, the artificial network displayed a balanced topology with neither excessive density nor fragmentation.

The breast-feeding network was denser and more complex, with a large interconnected core and multiple strongly associated clusters **(**Fig. 6B**).** Opportunistic and inflammation-associated genera including *Klebsiella*,* Bacteroides*,* and Clostridium sensu stricto 1* showed strong positive correlations, forming tightly linked modules, while *Streptococcus* displayed strong associations with *Faecalibacterium* and *Actinomyces*. Negative correlations involving *Bifidobacterium* and taxa such as *Escherichia–Shigella* and *Pseudomonas* were more frequent, indicating increased niche competition and greater heterogeneity in genus-level co-variation. This network structure reflects a less uniform and more competitive association pattern compared with artificial feeding.

The mixed fed network exhibited an intermediate topology that shared features of both breastfeeding and artificial feeding **(**Fig. 6C**)**. Strong positive associations were concentrated within *Ruminococcus* and *Lachnospiraceae*-related taxa, with *Blautia* acting as a highly connected node linking multiple genera. At the same time, moderate negative correlations were observed between *Bifidobacterium* and taxa such as *Ruminococcus gnavus* group and *Streptococcus*, indicating partial separation between beneficial and competing genera. The resulting network showed clustered connectivity without a single dominant core, consistent with a transitional community structure influenced by both feeding inputs.

## Discussion

The observed phylum level in this report shifts across feeding modes. The artificial feeding enriched in *Actinobacteria* at 48.5% with *Firmicutes* at 28.0% and *Proteobacteria* at 23.5% and no detectable *Bacteroidetes*, breastfeeding showing reduced *Actinobacteria* at 34.8% with higher *Proteobacteria* at 32.8% and detectable *Bacteroidetes* at 2.9%, and mixed feeding characterized by *Firmicutes* dominance at 54.8% with *Actinobacteria* at 18.0%. The phylum level patterns described here align with some published reports but contrast with others, reflecting well documented heterogeneity across infant microbiome studies. Consistent with these results, a markedly higher proportion of *Actinobacteria* was reported in 0–6-month-old infants fed goat milk formula (62.98%) compared with breastfed infants (43.74%)^51^. Breastfeeding can show lower *Actinobacteria* than formula feeding when the formula promotes bifidobacterial growth, while *Proteobacteria* can be relatively high in early neonatal samples due to normal early succession in an oxygen rich gut, and mixed feeding is often associated with higher *Firmicutes* consistent with faster microbiome maturation after reduced breast milk exposure^52^. The near absence of *Bacteroidetes* in the artificially fed group can reflect a combination of biological timing, cohort structure, and analytical sensitivity rather than a single cause. On the other hand, Several studies report the opposite direction to *Actinobacteria* enriched formula feeding, with formula fed infants showing higher *Bacteroidetes* and lower *Actinobacteria* than breastfed infants, including evidence that exclusive breastfeeding is associated with higher *bifidobacteria* and lower *Bacteroides* compared with formula feeding^53^ and a Korean cohort reporting lower *Actinobacteria* with higher *Firmicutes* and *Proteobacteria* in formula fed infants than in breastfed infants^54^. Therefore, the changes in infant gut microbiota composition cannot be attributed to feeding mode alone, as large comparative and meta-analytic studies illustrate that population background and study design strongly modify observed patterns through differences in age at sampling, geographic and cultural context, laboratory protocols, 16S rRNA regions, bioinformatics pipelines, and diet classification schemes, while birth mode and early antibiotic exposure consistently emerge as major co-drivers that can override or mask feeding-related effects by delaying *Bacteroides* colonization and reshaping phylum-level structure across cohorts^53,55^. We acknowledge the imbalance in group sizes (12 exclusively breastfed vs. 18 in each of the other groups) as a limitation of this exploratory study. Future studies with larger and balanced cohorts are needed to confirm these findings.

Alpha diversity in infancy often follows a feeding linked gradient but the direction depends on age and how studies define feeding. Alpha diversity patterns in early life are strongly shaped by feeding mode because diet provides the primary and continuous selective pressure on the infant gut microbiome, determining both substrate availability and colonization dynamics during a critical developmental window. Across 48 infant fecal samples in this study, with a median sequencing depth of approximately 45,000 high-quality reads per sample, alpha diversity differed significantly by feeding group. Observed richness was significant (*p* = 0.0038) and Shannon diversity was highly significant (*p* = 7.5 × 10⁻⁵) by Kruskal–Wallis testing. Diversity followed a stepwise pattern, with the lowest values in the artificial feeding group, higher values in the breastfeeding group, and the highest values with the widest spread in the mixed feeding group. Multiple cohort studies and meta-analyses demonstrate that feeding mode explains a substantial proportion of within-sample diversity variation, often exceeding the effects of delivery mode or infant sex, because breast milk, formula, and mixed feeding differ fundamentally in nutrient complexity, temporal stability, and exposure intensity^55^. Cohort and meta-analytic studies show that exclusive or predominant milk feeding supports a more constrained microbial community early in life, while partial exposure to multiple feeding sources increases substrate diversity and promotes higher richness and Shannon diversity^55^. Mixed feeding consistently shows the greatest dispersion in alpha diversity because it captures heterogeneous patterns of breast milk and formula intake, differences in timing and dose, and in some cohorts earlier or intermittent complementary feeding, all of which increase between-infant variability^56^. Longitudinal data further demonstrate that reduced breast milk exposure and dietary transitions are associated with increasing diversity and faster microbiome maturation, supporting the observation of higher and more variable diversity in mixed feeding compared with single feeding modes^23^.

Early feeding functions as a key determinant that moves the infant gut microbiome toward a characteristic community configuration. some diets push many infants toward a similar community profile while other diets allow multiple stable profiles which illustrate the infant heterogeneity in beta diversity. Breastfeeding clusters infant gut microbiomes in a distinct ordination region with lower between infant variability while artificial feeding shows broader dispersion and mixed feeding falls between and overlaps both. PERMANOVA supports differences for breastfeeding versus artificial feeding R² 0.142 p 0.001 and breastfeeding versus mixed feeding R² 0.103 p 0.001. Artificial versus mixed is not significant R² 0.042 p 0.083. Several studies report feeding related separation in beta diversity ordinations with breastfeeding linked to more clustered infant gut communities and formula exposure linked to broader dispersion across infants. This supports the idea that feeding shifts both the average community pattern and the degree of between infant spread in multivariate space^23^. A meta-analysis across seven cohorts reports consistent differences between exclusively breastfed and non-exclusively fed infants in overall community composition across populations, which implies systematic beta diversity differences rather than isolated taxon changes^57^. Cross population profiling across age shows that diet and environment contribute to measurable differences in community level distances in infancy^56^. These beta diversity differences likely happen because feeding changes what microbes can grow and how consistent the gut environment is across infants. Breast milk gives a more similar set of nutrients and bioactive compounds to many infants, so their gut communities tend to develop in a more similar way and cluster more tightly. Formula feeding varies more across products preparation and feeding patterns and it lacks some breast milk components that strongly select for specific microbes so infants can end up with many different community patterns which increases between infant dispersion. Mixed feeding often sits in between because it partly keeps the breast milk effect but also introduces the wider range seen with formula and these feeding effects combine with other strong drivers like age delivery mode antibiotics and home environment.

Our findings suggest feeding mode changes the infant gut microbiome mainly by shifting a few major groups and key genera rather than changing all taxa in the same way. Extreme *Firmicutes–Bacteroidetes* imbalance under artificial feeding has been reported previously in early infancy and is commonly interpreted as delayed or restricted colonization by *Bacteroidetes* rather than an active expansion of *Firmicutes*, especially when solid foods and complex polysaccharides are absent^58,59^. *Bifidobacterium* commonly dominates the infant gut, but its dominance is strongest with artificial feeding, where other bacteria grow less and the normal shift toward a more mature gut community happens more slowly^12,23^. The enrichment of *Bifidobacterium* in formula-fed infants (21.5%) compared to breastfed infants (where *Bifidobacterium* was not among the top four genera) contrasts with several Western cohorts where breastfeeding is typically associated with Bifidobacterium dominance. This discrepancy may reflect differences in formula composition available in the Egyptian market; some local formula products are supplemented with prebiotics (e.g., GOS/FOS) or probiotics that specifically support bifidobacterial growth. Unfortunately, detailed formula composition data were not available from all mothers, which we acknowledge as a limitation of the present study. Higher *Escherichia-Shigella* and *Enterobacter* in breastfed infants matches early colonization stages where facultative anaerobes use oxygen and help create conditions that later favor strict anaerobes^60,61^. While *Escherichia-Shigella* and *Enterobacter* are often considered opportunistic pathogens in certain clinical contexts (e.g., immunocompromised hosts, gastrointestinal disease), their enrichment in breastfed infants in this study is consistent with their ecological role as facultative anaerobes. These taxa reduce luminal oxygen levels, thereby facilitating subsequent colonization by strict anaerobes such as *Bifidobacterium* and *Bacteroides*, which are critical for gut maturation and immune development. Thus, their presence in early life is likely beneficial for normal gut succession rather than detrimental. Mixed feeding patterns with more *Streptococcus* plus *clostridial* and *Enterobacteriaceae* genera match longitudinal reports of a transition state where partial formula exposure increases diversity while some breast milk linked features remain^61^. Lower *Faecalibacterium* and *Subdoligranulum* under artificial feeding is also consistent with studies linking formula exposure to fewer fermenters and lower representation of butyrate related functions in infancy^62^. Our PICRUSt2-based functional prediction supported these taxonomic findings. Breastfed infants showed enrichment of KEGG Orthologs involved in human milk oligosaccharide degradation and butyrate production, consistent with a gut microbiota adapted to utilize breast milk components. In contrast, artificially fed infants exhibited a pro-inflammatory and oxidative stress-prone functional profile, including LPS biosynthesis and oxidative stress enzymes, which may reflect a less favorable gut environment. Mixed-fed infants displayed a hybrid functional landscape, combining methanogenesis, butyrate production, and bile salt hydrolase activity, indicating a more complex and potentially more mature metabolic capacity. These functional inferences align with the taxonomic shifts observed and highlight the potential metabolic consequences of different infant feeding practices^49^.

The strong positive correlation block among *Klebsiella*,* Enterobacter*,* Bacteroides*, and *Clostridium sensu stricto 1* in the artificial feeding group suggests a co-occurring opportunistic module where these taxa rise and fall together, which is a pattern often reported in the changed infant gut microbial profiles and in cohorts where *Enterobacteriaceae* overgrowth becomes common^63^. This clustering also matches earlier ecology studies showing that *Enterobacteriaceae* often increase under similar gut conditions, so their co-occurrence likely reflects shared environments such as higher oxygen levels and simple nutrients rather than direct cooperation^64^. The negative correlations in the breastfed group between *Bifidobacterium* and *Proteobacteria* such as *Escherichia Shigella* and *Pseudomonas* are consistent with a competitive pattern where *Bifidobacterium* dominance aligns with lower abundance of several facultative opportunists, although correlation alone cannot prove inhibition^65,66^. The mixed feeding infant shows a group of common anaerobic gut bacteria that tend to rise together, especially *Faecalibacterium* with *Blautia* and several *Lachnospiraceae* and *Ruminococcus* groups, which suggests a shift toward a more mature community that can support fermentation^23^. This matches the previous idea, where facultative bacteria often appear early and strict anaerobes increase later as gut oxygen drops and diet changes^67^. The weak to negative links between *Proteobacteria* such as *Enterobacter*,* EscherichiaShigella*,* Klebsiella*, and many anaerobic commensals fit this same idea because these groups often move in opposite directions across infant development rather than increasing together^68^. *Bifidobacterium* shows mixed links in this network, which fits mixed feeding as an intermediate exposure where the community can keep some early milk associated features while also moving toward a more anaerobe rich profile^69,70^.

The breastfed network shows a structured but not overcrowded pattern with central genera such as *Escherichia Shigella*,* Enterobacter*,* Streptococcus*,* Veillonella*, and *Bifidobacterium*, which matches reports that these genera are among the most abundant in infant stool and often vary together across early life samples^71^. The connected subcluster of anaerobic and fermentative taxa such as *Faecalibacterium* and *Lachnospiraceae* is also consistent with infant gut maturation where strict anaerobes increase as the gut becomes more anaerobic and as diet inputs broaden^72^. The artificial fed network being denser with tightly linked modules of *Klebsiella*,* Enterobacter*,* Bacteroides*, and *Clostridium sensu stricto 1* fits literature describing early life states enriched for these genera in some infants and settings, including strong enrichment of *Klebsiella*,* Enterobacter*, and *Clostridium sensu stricto 1* during the first months in several cohorts, which can appear as a co-varying opportunistic block rather than a single tax on signal^63^. The mixed fed network looks intermediate because it concentrates positive links among anaerobic commensals while showing weaker negative links between *Proteobacteria* and commensals, which fits the idea that mixed feeding often produces a transition profile rather than a separate stable type^72^.

In conclusion Feeding type shapes the infant gut microbiome in clear ways but it does not explain everything. Artificial feeding links to a less balanced community with lower diversity, wider spread in beta diversity, fewer fermenting bacteria, and tighter clusters of opportunistic genera that rise together. Breastfeeding links to a more organized and more similar microbiome across infants, with clearer clustering and a network that supports normal early development toward anaerobic fermenters. Mixed feeding does not form a separate microbiome type. It sits between breastfeeding and artificial feeding and shows the most variation because infants receive different amounts of breast milk and formula. Importantly, the variability observed across this study reinforces that feeding mode acts as a major but not exclusive driver of early microbiome development, with age, birth mode, antibiotic exposure, and population context strongly modifying observed patterns. These results support a model in which early diet influences both the direction and consistency of microbiome assembly, shaping not only which taxa are present but also how microbial communities organize and vary across infants during a critical developmental window.

## Data Availability

The sequencing data generated in this study were deposited in the NCBI Sequence Read Archive under Bio project accession number PRJNA1436626 and Bio samples: SAMN56477993 to SAMN56478021 and SAMN56614935 to SAMN56614953 http://www.ncbi.nlm.nih.gov/bioproject/1436626.
